# Molecular characterization and expression profile of the estrogen receptor α gene during different reproductive phases in *Monopterus albus*

**DOI:** 10.1038/srep27924

**Published:** 2016-06-13

**Authors:** Weidong Ding, Liping Cao, Zheming Cao, Xuwen Bing, Fazhen Zhao

**Affiliations:** 1 Wuxi Fisheries College, Nanjing Agricultural University, 9 East Shanshui Road, Wuxi 214081, China; 2 Key Laboratory of Freshwater Fisheries and Germplasm Resources Utilization, Ministry of Agriculture, Freshwater Fisheries Research Center, Chinese Academy of Fishery Sciences, Wuxi 214081,China; 3 Yellow Sea Fisheries Research Institute, Chinese Academy of Fishery Sciences, Qingdao, 266071, China

## Abstract

To understand the molecular mechanism of estrogen and to evaluate the role of the estrogen receptor in mediating estrogen action, the full-length cDNA of estrogen receptor α (ERα) was cloned from *Monopterus albus*, and its expression pattern and distribution were investigated. The ERα cDNA of *M. albus* includes an open reading frame of 1863 bp, a 140-bp 5’-untranslated region and a 797-bp 3’-untranslated region. Amino acid sequence homology analysis showed that the *Monopterus albus* ERα has a moderate degree of similarity with *Sebastes schlegelii, Zoarces viviparus* and *Haplochromis burtoni* (81.1%, 80.7% and 80.4%, respectively). Quantitative PCR results showed that the highest level of ERα expression was in the liver; the next highest level of expression was observed in the gonads, where it was expressed at high levels particularly in the ovary in developmental stages IV and V and in the testis in developmental stage II/III. Immunohistochemistry analysis showed that ERα was present as slender particles distributed mainly in the membranes of spermatocytes and oocytes in the testis and ovary, whereas no positive signal was observed in the cytoplasm of sperm cells. This report describes the first molecular characterization of full-length ERα and its tissue-specific distribution in *M. albus.*

In vertebrates, the genomic actions of the sex steroid 17 β-estradiol (E2) are mediated through nuclear estrogen receptors (ERs)^1^,^2^. ERs can regulate the transcription of target genes, promote the formation of heterodimers and regulate the physiological functions of E2 by binding to DNA response elements with high affinity. There are two types of ER in teleost fish, one of which is ERα; knockout of ERα in male mice can lead to infertility. The ERα structure is characterized by functional domains A–F. The N-terminal A/B domains show high interspecific variability and transactivate target gene transcription directly through the AF-1 domain or after interaction with co-activators. The C domain or DNA-binding domain is highly conserved and contains two zinc fingers, which are involved in specific DNA binding and receptor dimerization. The D or hinge domain permits an interaction between the C domain and DNA owing to its flexible structure, affects DNA binding properties and might anchor some co-repressor proteins. The E/F or ligand-binding domains (LBDs) contribute to dimerization and play a pivotal role through the second ligand-activated transactivation domain (AF-2). Studies of fish gonads in aquaculture have shown the presence of the ER with a high affinity for E2. ER mRNA has been found in the male gonads of a variety of fish^3^,^4^,^5^,^6^,^7^, including the Atlantic croaker *Micropogonias undulatus*^8^, *Tilapia* spp.^9^, Nile tilapia *Oreochromis niloticus*^10^, channel catfish *Ictalurus punctatus*^11^, zebrafish *Danio rerio*^12^, Atlantic croaker *Micropogonias undulatus*^13^, gilthead bream *Sparus aurata*^4^ and rainbow trout *Oncorhynchus mykiss*^14^. Hawkins *et al*.^8^ found three types of ER in the testis of *M. undulatus*. ERα was also found in the testicular tissues of *O. mykiss*^14^, and ERα mRNA expression was detected in these tissues. A study of goldfish *Carassius auratus* by Choi and Habibi showed that the mRNA of ERα was expressed mainly in the pituitary glands of females and males^15^. Immunohistochemistry analyses showed that ERα mRNA was expressed in the ovary and testis of the mullet *Mugil cephalus*^16^, indicating that ERs play an important role in mediating the physiological effects of estradiol E2. In addition, the ER was found to be expressed in the spleen and head kidney of *O. mykiss*, which are the main organs in the fish immune system^17^, although some other immune cells in fish can express ER mRNA, including *S. aurata* endothelial cells^18^, macrophages and lymphocytes^19^ and *O. mykiss* leukocytes^20^, suggesting that ERs might play a role in the immune function of teleost fish.

The rice field eel lives mainly in Asia, and only *Monopterus albus*, which has a high nutritional value, is found in China. Since the culture of *M. albus* in the beginning of the 1970s, a large number of immature wild *M. albus* have been captured. Unfortunately, environmental pollution has led to the dwindling resources of wild *M. albus*; thus, there is a severe shortage of breeding individuals. A sex reversal phenomenon in the developmental cycle of *M. albus* was first discovered by Liu *et al*.^21^. Subsequently, Xiao *et al*. studied the development and differentiation of *M. albus* gonads in detail^22^,^23^. They found that *M. albus* develops first into a female. After sexual maturity and spawning, male germ cells start to develop, and the fish enter a stage of intersexuality before they develop gradually into males. Sex-determining genes in *M. albus* and the regulation of sex reversal at the gene level have been the focus of research studies. Sex determination is a complex regulatory process involving multiple genes at different times and locations. However, the cloning of ERα and details of its structural characteristics in *M. albus* have not been reported. In this study, we cloned and characterized *M. albus* ERα mRNA using RT-PCR and investigated its tissue distribution and chromosomal location.

## Results

### Cloning ERα cDNA and the deduced amino acid sequence

**Figure 1:**
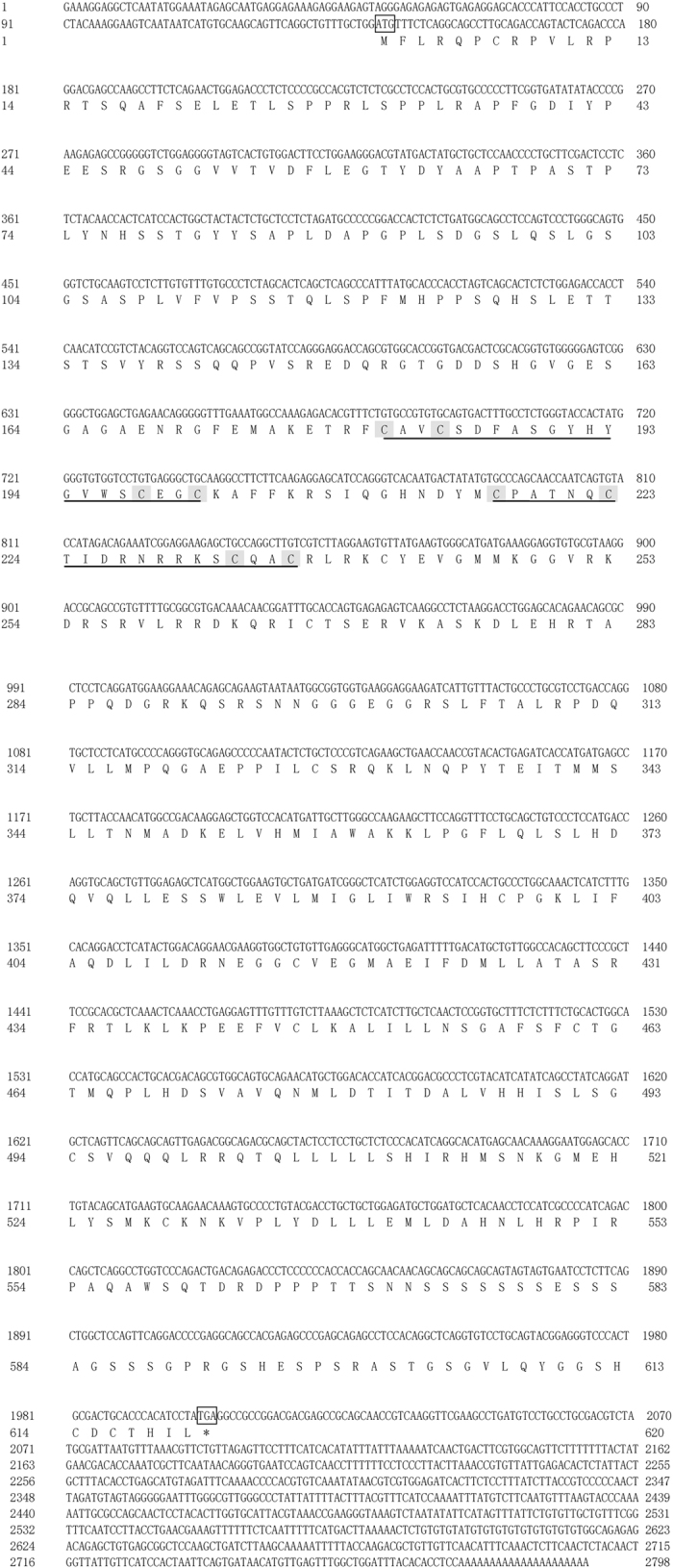
Nucleotide and deduced amino acid sequence of *Monopertus albus* ER islolated from gonad. Two zinc-finger motifs in DNA binding domain were underlined and eight cysteines in ths same domain were also shaded. The initiation codon and termination codon were boxed.

The full-length ERα cDNA sequence obtained from *M. albus* ovary consisted of 2798 bp, including the poly (A) tail (Fig. 1). The sequence included an open reading frame of 1863 bp, a 140-bp 5′ untranslated region (5′UTR) and a 797-bp 3′UTR. The open reading frame encoded a putative protein of 620 amino acid residues with a calculated molecular mass of ~58.3 kDa. The putative polyadenylation signal TATAAA was located 15 bases upstream of the poly (A) tail.

**Figure 2:**
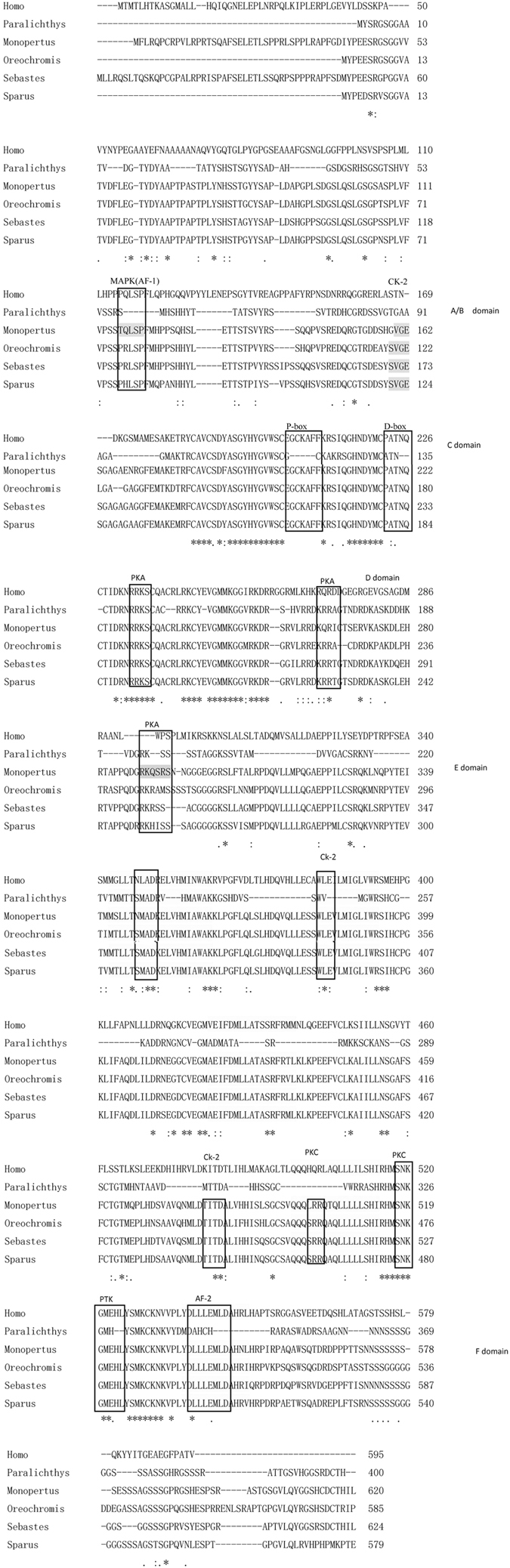
Amino acid alignment of *Monopertus albus* ERα with other ERα from diffierent teleosts and mammalians. Asterisks (*) and DOTS (:) marked for completely conserved and conserved amino acids, respectively. The functional domains (A/B,C [DNA-binding domain], D,E [ligand-binding domain], and F) and the P- and D-box in C domain, as well as the activation domains (AF-1 and AF-2) in the A/B and D domain, respectively, are indicated. Eight cysteines in the C domain were underlined. Potential phosphorylation sites for MAPK, PKA, PKC, CK-2 and PTK are boxed. MAPK: mitogen-activated protein kinase; PKA: protein kinase A; PKC: protein kinase C; CK-2: casein-kinase II; PTK: protein tyrosine kinsase. In the E/F domain,the helices surrounding the ligand binding cavity are in reverse type.

The deduced amino acid sequence was aligned by Mega software. Analysis showed that the ERα from *M. albus* shares moderate degrees of homology with the ERα proteins from *Sebastes schlegeli, Zoarces viviparus* and *Haplochromis burtoni* (with identities of 81.1%, 80.7% and 80.4%, respectively). Similar to all nuclear receptors reported to date, ERα consists of six distinct domains, including a variable A/B domain at the N terminus, a highly conserved C domain (a DNA-binding domain), a D domain (hinge region) and an E domain (an LBD). As shown in Fig. 2, there is a consensus motif p-x (1, 2)-sp, which is a mitogen-activated protein kinase phosphorylation site that is recognized as the main component of the ligand-independent transactivation function motif AF-1 in the A/B domain region^24^. In addition to the conserved C domain, there are three other conserved regions, including eight cysteine residues located in two zinc-finger motifs, a D-box (EGCKAFF) and a P-box (PATNQ). A protein kinase C phosphorylation site, a tyrosine kinase phosphorylation site and a ligand-dependent transactivation function motif (AF-2), present in teleosts and mammals, were also conserved. Several potential phosphorylation sites for casein kinase II, protein kinase A and protein kinase C are also conserved in teleosts.

### Phylogenetic analysis of ERα

**Figure 3:**
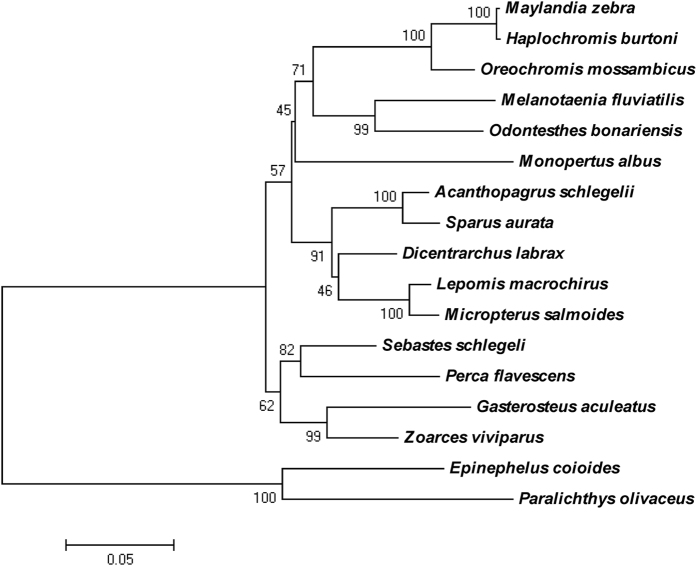
Phylogenetic tree based on amino acid sequences for ERα proteins. The tree was built using Mega 5.0 software the clustal method was used to perform multiple sequence alignment. The following full length aminoacid sequences were used: *Maylandia zebra*, XP_004541776.1; *Haplochromis burtoni*, AAR82891.1; *Oreochromis mossambicus*, CAK95869.1; *Melanotaenia fluviatilis*, ADF87494.1; *Odontesthes bonariensis*, ABY19510.1; *Acanthopagrus schlegelii*, AAL82743.1; *Sparus aurata*, CAB51479.1; *Dicentrarchus labrax*, CAD43599.1; *Lepomis macrochirus*, ABP96712.1; *Micropterus salmoides*, AF253062_1; *Sebastes schlegelii*, ACN39246.2; *Perca flavescens*, ABL64073.1; *Gasterosteus aculeatus*, NP_001254601.1; *Zoarces viviparous*, AAO66473.1; *Epinephelus coioides*, ADK90033.1; *Paralichthys olivaceus*, BAB85622.1.

Phylogenetic analysis of the inferred amino acid sequence of ERα was used to determine its evolutionary position. The analysis revealed that the ERα of *M. albus* is closely related to *Maylandia zebra, H. burtoni, Oreochromis mossambicus, Melanotaenia fluviatilis* and *Odontesthes bonariensis*, as shown in Fig. 3.

### ERα expression patterns in different tissues

**Figure 4:**
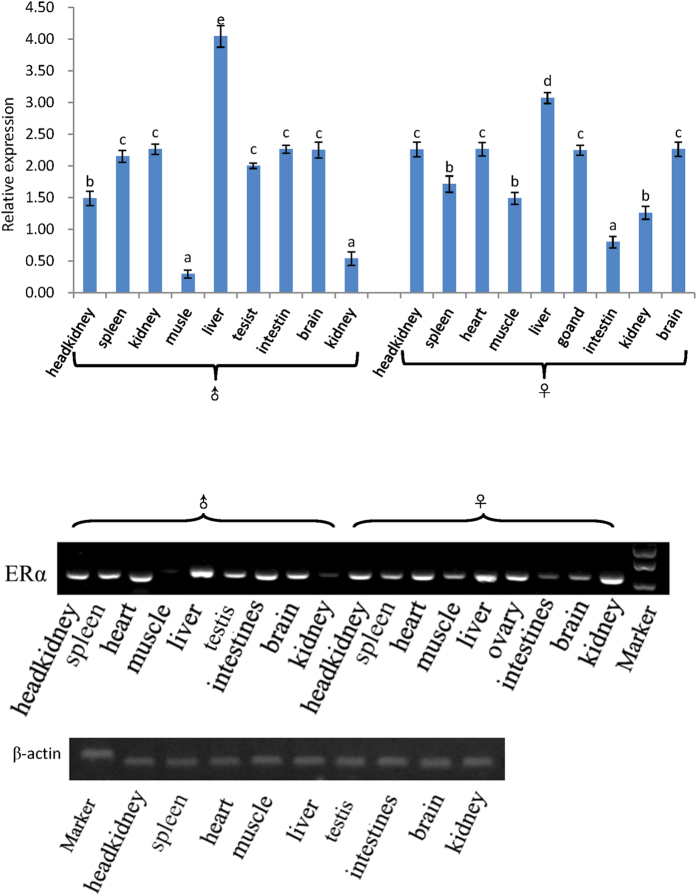
Expression of ERα measured by real-time PCR in different tissues of ricefield eel. The results are presented as a mean(n = 6) ± standard deviation of the means(SD). The expressin is normalized to β-actin.

Specific primers were designed according to the conserved regions of the ERα mRNA sequences from *M. albus* and other fish species in GenBank to detect its expression level. A fragment with a size of 253 bp could be amplified with the primer pair ERα F4 and R4. β-Actin was used as a reference gene for detection using quantitative fluorescent PCR. ERα expression was determined in nine different tissues from *M. albus* males and females, including the testis, liver, spleen, kidney, head kidney, intestine, heart, brain and muscle. The ERα gene is expressed in all nine tissues (Fig. 4) but at different levels. The expression levels were low in the muscle and kidney of *M. albus* males and in the intestine and kidney of females, whereas the expression levels were highest in the livers of *M. albus* males and females.

### ERα mRNA expression in the gonad during the reproductive cycle

**Figure 5:**
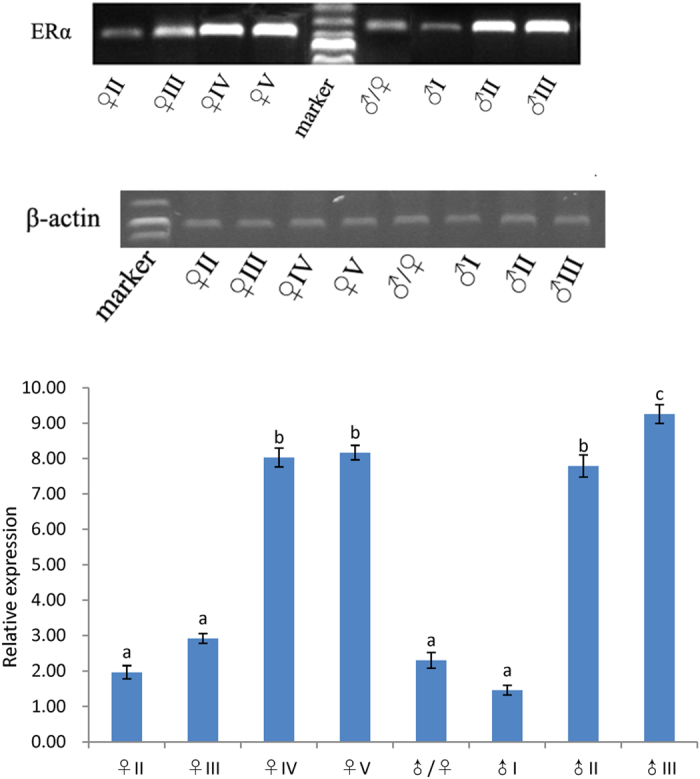
Expression of ERα measured by real-time PCR during sex development of ricefield eel. The results are presented as a mean(n = 6) ± standard deviation of the means(SD). The expressin is normalized to β-actin.

Real-time RT-PCR was used to evaluate the ERα mRNA expression profiles in the ovary and testis during the reproductive cycle. β-actin was used to normalize the ERα mRNA products to obtain quantitative results. ERα was expressed at high levels in the ovary mainly in developmental stages IV and V and in the testis in developmental stage II/III (Fig. 5). The levels of ERα expression were not high in either the ovary in developmental stage III or the testis in developmental stage I; ERα expression was also not high in the intersex stage.

### Immunohistochemistry

**Figure 6:**
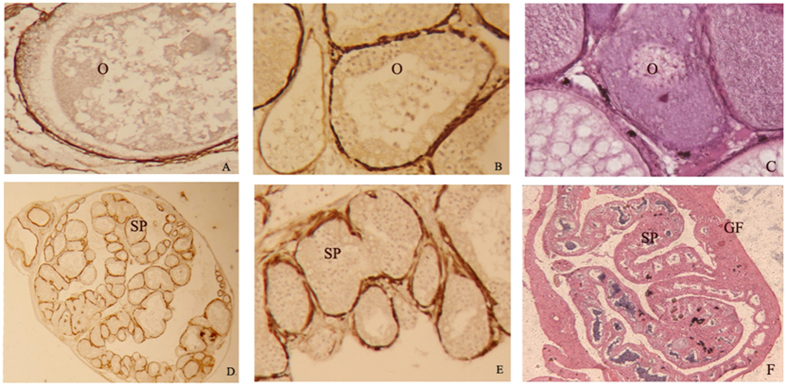
Estrogen receptor alpha immunostaining in gonad of *Monopterus albus*, (**A**–**C**) are ovary, (**D**–**F**) are testis. No immunostaining is seeing in negative control (**C**,**F**). O: ovary, GF gonopores, SP: spematophore.

A sequence with high antigenicity to ERα was obtained by sequence analysis and was then cloned into a prokaryotic expression vector. Polyclonal antibodies were then prepared and used to perform immunohistochemical analysis of the gonadal tissues of *M. albus*. As shown in Fig. 6A,B,D,E, immunostaining revealed precipitates with shades ranging from brown to brownish-yellow in sections of the *M. albus* testis and ovary, whereas the background was colorless or light brown. Positive signals were observed mainly in the cell membranes of spermatocytes and oocytes in the testis and appeared to consist of more slender particles; no positive signal was observed in the cytoplasm of sperm cells. Sections of the gonads of the control group were counterstained bluish-purple by hematoxylin (Fig. 6C,F), and no positive signals of brown particles were observed.

### *In situ* hybridization

**Figure 7:**
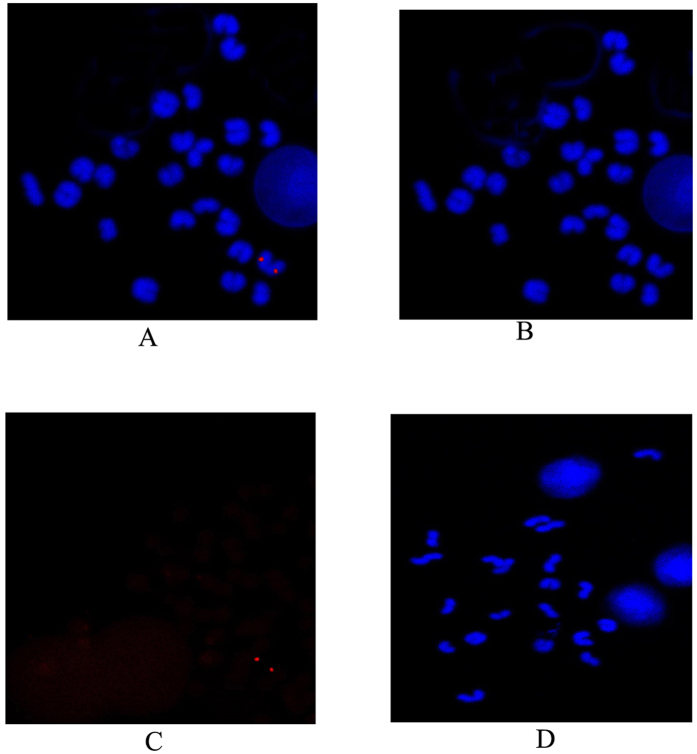
Chromosomal localization of *Minipertunubal albus* ERα on metaphase chromosomes by FISH. Nucleus and metaphase chromosomes were stained with DAPI (green **A**,**B**,**D**). (**A**) shows a typical metaphase chromosomes hybridiaed with a ERα probe (red). (**D**) shows negative control. Scale bar = 5 μm.

Good-quality metaphase chromosomes were obtained in prepared *M. albus* head kidney tissues, as shown in Fig. 7D, using an intraperitoneal injection of phytohemagglutinin (Biosun, Shanghai, China). Probes were synthesized and used for the effective localization of ERα on the metaphase chromosomes using FISH, as shown in Fig. 7A–C. Positive hybridization signals were observed in the autosomes, but no signal was observed in the control.

## Discussion

ER and estrogen play very important roles in the development of the reproductive system in animals. Understanding and localizing the distribution of ERs in aquatic animals will aid in understanding the role of estrogen in sexual development. Thus far, there has been no report of either the distribution or the role of the ER in *M. albus*. In this study, the full-length cDNA of ERα from the gonads of *M. albus* was cloned using PCR, and the location of expressed ERα in the gonads was investigated. Thus, the potential role of ERα in *M. albus* gonadal development can be understood. Estrogen has a variety of physiological functions and is involved in regulating animal reproduction, metabolism, homeostasis, cell proliferation, differentiation, apoptosis and inflammation. The ER is part of the cellular machinery needed to ensure that estrogen performs these functions. In the first study of the ER, ERα was isolated and purified from human breast cancer cells in 1986 by Green *et al*.^25^. In the study of teleost fish, the first ERα was isolated from *O. mykiss*^26^. Thus far, several fish species have been used for the study of ERα gene cloning and its expression in tissues. In this study, a primer pair was designed according to the gene sequence of fish ERα, and the full-length cDNA of the ERα gene was cloned from the ovary of *M. albus* using RT-PCR and random amplification of cDNA ends (RACE) techniques. The full-length cDNA of the gene was 2798 bp. BLAST analysis and alignment of the amino acid sequences indicated that the sequence had a high level of homology with the ERα genes from *S. schlegelii*, *Z. viviparus* and *H. burtoni*, confirming that the cDNA cloned was the *M. albus* ERα gene. Alignment of the amino acid sequence of the gene and sequences from other fish species revealed that the ERα of *M. albus* contained typical A/B, C, D, E and F molecular domains of ERα (Fig. 3). Compared with other teleost fish, the A/B domain of the *M. albus* ERα was short, and the C and E domains were less conserved and rich in serine and proline residues. Phosphorylation sites recognized by mitogen-activated protein kinase (MAPK) are present in the A/B domain of ERα, indicating that ERα might activate the MAPK pathway^27^. The AF-2 Activation domain (DLLLEML) occurred between amino acid residues 536 and 545 of the LBD domain, indicating that transcriptional activity was dependent on ligand binding^28^. Sequence analysis revealed a potential activator protein-binding site (TGACTAT) located between amino acid residues 326 and 333, which was similar to mammals, and five tyrosine residues existed in the LBD domain of ERα, which was highly conserved in all teleost fish. Tyrosine 454 was conserved in all vertebrates, and the binding of ligands to tyrosine residues could lead to phosphorylation^15^.

In this study, we investigated the expression pattern of ERα mRNA in different tissues and developmental stages of *M. albus*. ERα was expressed in most tissues of *M. albus* males and females, with the highest expression level in the liver. ERα mRNA is likely to be expressed in different tissues and organs of fish but it is expressed mainly in the gonads, liver and pituitary gland. Other studies have shown that ERα mRNA expression level in goldfish was highest in the pituitary gland^15^. In contrast, in the half-smooth tongue sole (*Cynoglossus semilaevis*), ERα mRNA was expressed mainly in the liver, which was considered to be related to metabolism^29^. Studies on *S. aurata*^30^ showed that ERα was expressed mainly in the liver and pituitary gland. Studies on *O. mykiss*^31^ indicated that the very highest ERα1 and ERα2 expression levels were in the liver, with the second highest levels in the testis and the lowest levels in the stomach. Similar results were obtained in this study, where the highest ERα expression level was in the livers of *M. albus* males and females, with high levels in the testis and ovary, findings that are consistent with the conclusion that the liver is the main organ for ERα expression. We suggest that the liver is the main site in fish for vitellogenin synthesis. Estrogen first binds to the ER, then to the estrogen response element, and eventually initiates the transcriptional regulation of the vitellogenin gene^32^. The patterns of ERα expression in different developmental stages of *M. albus* were investigated in this study. The levels of ERα expression were relatively high in the testis in stages II and III and in the ovary in stages IV and V. Although ERα was expressed in the gonads at other stages, the expression levels were low, suggesting that the ER plays an important role in the maintenance of gonadal function. This finding was consistent with the pattern of ERα expression in goldfish during the process of ovarian development; the ERα expression level was low during early gonadal development in goldfish, and the level was highest when the gonads were mature^33^.

In the past few years, many researchers have reported a two-way interaction between neutral steroid hormones and the immune system^34^ in vertebrates, including fish^35^,^36^. Studies have demonstrated the existence of such an interaction in teleost fish. On one hand, genes encoding immune-related proteins also encode hormone receptor-related proteins. A large number of immune-related proteins, such as lectins^37^, lysozyme, hepcidin and complement components^38^,^39^, including ERα, have been identified in fish gonads. Massartr found that ERα was expressed in the immune organs of *O. mykis*s^40^. On the other hand, a growing number of studies have shown that sex steroid hormones have an effect on the fish immune system^41^,^42^. For example, complement activity and IgM activity were suppressed in *S. auratus* at 7 days after treatment with estrogen^43^. In addition, treatment with estrogen for 14 days could adjust the expression of MHC1, chemotaxin and other immune-related complement component genes in *O. mykiss*^44^. In this study, ERα was expressed in immune-related *M. albus* tissues, including the spleen, kidney and head kidney, demonstrating that the interactions between sex steroid hormones and the immune system exist.

ERα expression in *M. albus* was investigated using immunohistochemistry. The results of localization studies revealed positive reactions of ERα in sections of *M. albus* ovarian and testicular tissues. The nucleoli of spermatogonia and nuclei of spermatocytes in different developmental stages and sperm cells in different stages showed negative immunoreactivity, while interstitial cells between the lobules of testis all showed positive immunoreactivity. The membranes and nuclei of oocytes in ovaries in different stages all showed positive immunoreactivity. These results were similar to those for ERα expression in other animals. Madhabananda^45^ studied ERα expression in the rat ovary and demonstrated that ERα was expressed in the cal cells, interstitial cells and germinal epithelium, suggesting that ERα can be expressed in the membranes of rat oocytes. Fang *et al*.^46^ performed an immunohistochemical test of ERα in the mullet testis and found that ERα was located mainly in the spermatogonia, primary spermatocytes and secondary spermatocytes, while nucleoplasm, sperm cells and sperm showed negative immunoactivity. Bouma *et al*.^47^ reported that ERα was expressed only in fibroblasts (the precursor cell of interstitial cells) in the testicular interstitial fibroblasts of *O. mykiss* and reported that one of the functions of estrogen was to induce the precursor cells to differentiate into mature interstitial cells.

*In situ* hybridization showed that *M. albus* chromosomes had 12 pairs of homologous chromosomes and no obvious secondary constrictions, satellites or telocentric centromeres. The ER gene probe synthesized by PCR could detect the presence of the ER gene on the chromosome. Fish hold a critical position in the phylogeny of vertebrates. Members of the order synbranchiformes (an order of eel-like actinopterygian fish, commonly called swamp eels) are among the most highly specialized fish species. *M. albus* is the only representative species of the genus Monopterus in the family Synbranchidae of the order Synbranchiformes. Localization of the ER gene on the chromosomes investigated in this study will increase the number of known genetic markers on *M. albus* chromosomes, which will help to distinguish and identify each chromosome and to establish high-precision genetic mapping of *M. albus* based upon information concerning the chromosomes and the genome. Thus, the genetic mechanism of natural sex reversal and the sources and evolutionary mechanism of *M. albus* chromosomes will be revealed at the cellular and molecular levels.

In conclusion, the full-length cDNA of ERα was first cloned from *M. albus* gonads in this study, and its expression pattern and distribution were investigated. The full-length cDNA sequence of *M. albus* ERα was 2798 bp, which encoded a 58.3-kDa protein consisting of 620 amino acid residues. Amino acid homology analysis showed that it has a high degree of similarity with *S. schlegelii*, *Z. viviparus*, and *H. burtoni*. Sequence analysis showed that ERα consists of six domains, including a variable A/B domain and C, D and E domains. The ERα expression pattern was studied using qRT-PCR, and the results showed that it was expressed in different tissues, but mainly in the liver. However, the expression level in the gonads was the second highest and reached a maximum during gonadal maturation. Immunohistochemistry showed that ERα was distributed mainly in the cell membranes of spermatocytes and oocytes, and no positive signal was observed in the cytoplasm of sperm cells.

## Experimental Section

### Animals

All experiments were approved by the Institutional Animal Care and Use Committee of the Ministry of Freshwater Fisheries Research Center of the Chinese Academy of Fishery Sciences and were undertaken in accordance with the national legislation for fish welfare established by the Ministry of Science and Technology of the People’s Republic of China. All *M. albus* ranging in length from 12–75 cm were sampled from a population kept at the Wuxi Fishery Fish Research Center, China. Different tissues were collected for RNA extraction, and gonad tissues were used for histological examination. In this study, all females were <27 cm in length, all males were >40 cm in length and intersexual fish were 26–38 cm in length.

### RNA extraction

Total RNA was extracted from the liver and other tissues using RNAiso Plus (Takara, Dalian, China), as recommended by the manufacturer. Briefly, after 0.1-μg tissue samples were ground and pulverized, 1000 μl of RNAiso Plus was added with repetitive pipetting until the tissues were lysed completely. Next, 0.2 volumes of chloroform was added, and the mixture was left at room temperature for 5 min and then centrifuged at 12,000 *g* for 5 min. The supernatant was removed, and an equal volume of anhydrous isopropanol was added to precipitate the RNA. The absorptions at 260 nm (*A*_260_) and at 280 nm (*A*_280_) were measured with a spectrophotometer, and the *A*_260_/*A*_280_ ratio was used to assess RNA quality before the samples were stored at −70 °C.

### Isolation of ERα cDNA

The ERα primers were designed based on a published partial ER sequence (GenBank accession number: AY686635.1) of *M. albus*. Samples (1 μg) of total RNA from liver, brain, gonad and other tissues were retrotranscribed using a cDNA synthesis kit (TakaraBio Inc., Dalian, China) with oligo (dT_18_) primer. PCR used 2 μl of synthesized cDNA as a template. All reactions contained 25 μl of 200 nM ERα F1 and R1 primers (listed in Table 1), 200 μM of each dNTP, 2 mM MgCl_2_ and 1.2 U of rTaq DNA polymerase (Takara Bio Inc.). The amplification protocol was as follows: predenaturation at 95 °C for 30 s, 30 cycles of denaturation at 95 °C for 10 s, annealing at 57 °C for 30 s and a final elongation step at 72 °C for 45 s. All PCR products were electrophoresed in 1.5% (w/v) agarose gel stained with ethidium bromide to estimate the molecular mass of the amplicons. The target band of predicted size was gel-purified using a Gel Extraction kit (Takara Bio Inc.), cloned into the pMD-18-T vector (Takara Bio Inc.) and sequenced by Biosun Biotech (Shanghai, China). All experiments were performed in triplicate.

The 5′ and 3′ ends of the ERα cDNA were obtained according to the manufacturer’s instructions for the RACE kit (Takara). Four gene-specific primers (see Table 1) were designed for the RACE reaction. The PCR products were subjected to electrophoresis in a 1% (w/v) agarose gel and were purified using a Gel Purification kit (Takara). The purified product was recovered, cloned into the pMD-18-T vector and then sequenced by Biosun Biotech (Shanghai, China).

### Phylogenetic analysis

The full-length ERα sequence was analyzed with BLAST software on the NCBI website, and the sequence was aligned in GenBank. The amino acid sequences of ERα from other species, which share high degrees of amino acid similarity with ERα, were downloaded and analyzed using Clustal W software. A phylogenetic tree of these sequences was constructed with the neighbor-joining method. The sequences used for the phylogenetic analysis were obtained from: *Maylandia zebra*, XP_004541776.1; *Haplochromis burtoni*, AAR82891.1; *Oreochromis mossambicus*, CAK95869.1; *Melanotaenia fluviatilis*, ADF87494.1; *Odontesthes bonariensis*, ABY19510.1; *Acanthopagrus schlegelii*, AAL82743.1; *Sparus aurata*, CAB51479.1; *Dicentrarchus labrax*, CAD43599.1; *Lepomis macrochirus*, ABP96712.1; *Micropterus salmoides*, AF253062_1; *Sebastes schlegelii*, ACN39246.2; *Perca flavescens*, ABL64073.1; *Gasterosteus aculeatus*, NP_001254601.1; *Zoarces viviparus*, AAO66473.1; *Epinephelus coioides*, ADK90033.1; and *Paralichthys olivaceus*, BAB85622.1.

### ERα expression pattern analysis throughout sex reversal

Fluorescent quantitative real-time (RT) PCR (qRT-PCR) was used to analyze the ERα expression pattern during sex development. Specific primers for ERα F4 and R4 were designed for gene expression analysis during sex development based upon the complete cDNA sequences of ERα genes. The β-actin gene (actin-F and actin-R) was selected as the internal control gene, and the length of the fragment was 204 bp. RT-PCR used a 7500 RT-PCR system (Applied Biosystems, USA). The reaction contained 12.5 ± 1 μl of SYBR Premix Ex Taq, 0.5 ± 1 μl of each primer, 2 ± 1 μl of DNA template and 9.5 ± l μl of sterile water in a total volume of 25 ± 1 μl. The amplification protocol was as follows: predenaturation at 95 °C for 30 s, 40 cycles of denaturation at 95 °C for 10 s and annealing at 60 °C for 30 s. All experiments were performed in triplicate. The results were analyzed using 7500 System SDS Software. A negative control was used in all experiments to exclude false-positive results. Each reaction (10 ± 1 μl) was electrophoresed in a 1.5% (w/v) agarose gel. The relative expression levels of genes were calculated using the 2^−ΔΔ*C*T^ method. Statistical analysis with one-way analysis of variance (ANOVA) was performed using SPSS 15.0 software (SPSS, Chicago, IL, USA).

### Immunohistochemistry

Immunohistochemical reactions were measured using the SuperPicureTM method. Mouse antibodies against the *M. albus* estrogen receptor were polyclonal antibodies generated in our laboratory, and a 1:100 dilution was used. A 1:150 dilution of goat anti-mouse antibodies labeled with horseradish peroxidase (Beyotime Biotechnology Institute, Jiangsu, China) was used. A 3,3′-diaminobenzidine (DAB) color development kit was purchased from Beyotime Biotech Reagent Co. Normal mouse serum was incubated to replace the primary antibody on the control slide; the hematoxylin counterstain was negative.

### Detection of chromosomal locations with fluorescence *in situ* hybridization (FISH)

Probes were synthesized according to the manufacturer’s instructions for the PCR DIG Probe Synthesis kit (Roche, Germany). Briefly, the forward and reverse primers, which were designed using the method of Lou^48^, were ERα F5 and R5, as listed in Table 1. The protocol for PCR amplification was 35 cycles at 98 °C for 10 s, 60 °C for 15 s, 68 °C for 4 min and a final elongation step at 68 °C for 10 min. All PCR products were resolved on 1% agarose gels. The amplified products were sequenced and confirmed by BiosunBiotech Co. Metaphase chromosomes were prepared with the method of Cao^49^, and *in situ* hybridization was performed using the HNPP Fluorescent Detection Set (Roche, Germany) according to the manufacturer’s instructions.

### Preparation of chromosome specimens

Slides with the prepared chromosomes were baked at 60 °C. The appropriate amount of RNase (100 μg/mL, dissolved in 2× saline sodium citrate [SSC]) was added dropwise to the slides, which were then incubated in a water bath at 37 °C for 1 h. The samples were washed with 2× SSC at room temperature and then dehydrated sequentially with 70%, 85%, 90% and 100% (v/v) ethanol. The samples were digested with an appropriate amount of pepsin (0.01% (w/v) dissolved in 0.01 M HCl in a water bath at 37 °C for 10 min. They were then washed with PBS (containing 50 mM MgCl_2_ and 1% (v/v) formaldehyde) and dehydrated sequentially again with 70%, 85%, 90% and 100% ethanol before they were dried at room temperature.

### Hybridization *in situ*

Hybridization solution (total volume 20 μL) was added dropwise onto the hybridization zone. The probe hybridization solution (containing 50% deionized formamide, 2× SSC, 10% (w/v) dextran sulfate, 50 mM sodium phosphate and 2.5 ng of labeled DNA probe for ERα was heated at 80 °C for 5 min to denature the DNA and then chilled on ice for 10 min. A coverslip was added to the slide, which was then placed into a wet box and left overnight in darkness at 37 °C.

### Elution and fluorescence detection

After hybridization, the slides were placed into 2×SSC, 50% (v/v) formamide for 15 min and then into 2×SSC solution for 15 min at 45 °C. The slides were washed thoroughly with washing buffer (Roche, USA) and blocked with blocking solution (Roche). An alkaline-phosphatase-conjugated anti-digoxin antibody (Roche) was added dropwise to the slides, which were then incubated at 37 °C for 60 min. Each slide was then washed three times, 5 min each time, with washing buffer (Roche) and three times, 5 min each time, with detection buffer (Roche). HNPP/Fast Red TR Mix (Roche) was added, and the samples were incubated at 20 °C for 30 min. This process was repeated twice, and the samples were rinsed with washing buffer for 10 min between reactions. The slides were soaked in double-distilled water for 10 min to stop the reaction. The samples were counterstained with 20 μL of 4′,6-diamidino-2-phenylindole (0.02 mg/mL), and a coverslip was placed onto the slide. The stained samples were examined under a fluorescence microscope.

### Image detection and analysis

The hybridization signals on the chromosome slides were observed in darkness using an Olympus BX51 fluorescence microscope. The images were captured with a SenSys CCD camera controlled using FISH view 5.5 software (Applied Spectral Imaging, Inc., USA).

### Statistical analysis

All data are expressed as the means ± standard deviations (mean ± SD). One-way ANOVA was used to conduct mean significance tests between each group. Significance for all statistical comparisons was set at p < 0.05. All data processing and analyses were performed using the SPSS15.0 statistical package.

## Additional Information

**How to cite this article**: Ding, W. *et al*. Molecular characterization and expression profile of the estrogen receptor α gene during different reproductive phases in *Monopterus albus*. *Sci. Rep.* **6**, 27924; doi: 10.1038/srep27924 (2016).

## Figures and Tables

**Table 1:** Sequences of PCR primers.

No	Purpose	primer	*5′ to 3′ sequence*	
1	Partail cDNA PCR	ERα F1	TATGTGCCCAGCAACCAATC	
		ERα R1	CCTTCGTTCCTGTCCAGTATG	
2	5′ RACE PCR	ERα R2	CCTCCGATTTCTGTCTATGGTA	
		ERα R3	GCCCACTTCATAACACTTCCTA	
3	3′ RACE PCR	ERα F2	TGAGATCTTCGACATGCTGC	
		ERα F3	TGGCTGTGTTGAGGGCATGG	
4	Quantitative RT-PCR	ERα F4	GCCAGGCTTGTCGTCTTAGG	
		ERα R4	TCCTTCACCACCGCCATTA	
5	β-Actin	F1	ACTTTGAGCAAGAAATGGGAACT	
		R1	GGACTCAGGGCAACGGAAC	
6	ISH probe	ERα F5	GACTATGCTGCTCCAACCC	
		ERα R5	CCCAAATTCCCCCTACTACA	
